# Molecular characterization of two A-type P450s, *WsCYP98A* and *WsCYP76A* from *Withania somnifera* (L.) Dunal: expression analysis and withanolide accumulation in response to exogenous elicitations

**DOI:** 10.1186/s12896-014-0089-5

**Published:** 2014-11-23

**Authors:** Satiander Rana, Wajid Waheed Bhat, Niha Dhar, Shahzad A Pandith, Sumeer Razdan, Ram Vishwakarma, Surrinder K Lattoo

**Affiliations:** Plant Biotechnology Division, CSIR-Indian Institute of Integrative Medicine, Canal Road, Jammu, Tawi-180001 India; Medicinal Chemistry Division, CSIR-Indian Institute of Integrative Medicine, Canal Road, Jammu, Tawi-180001 India

**Keywords:** *Withania somnifera*, Withanolides, Cytochrome P450 monooxygenase, mRNA, Phytohormones, *E.coli*

## Abstract

**Background:**

Pharmacological investigations position withanolides as important bioactive molecules demanding their enhanced production. Therefore, one of the pivotal aims has been to gain knowledge about complete biosynthesis of withanolides in terms of enzymatic and regulatory genes of the pathway. However, the pathway remains elusive at the molecular level. P450s monooxygenases play a crucial role in secondary metabolism and predominantly help in functionalizing molecule core structures including withanolides.

**Results:**

In an endeavor towards identification and characterization of different P450s, we here describe molecular cloning, characterization and expression analysis of two A-type P450s, *WsCYP98A* and *WsCYP76A* from *Withania somnifera*. Full length cDNAs of *WsCYP98A* and *WsCYP76A* have open reading frames of 1536 and 1545 bp encoding 511 (58.0 kDa) and 515 (58.7 kDa) amino acid residues, respectively. Entire coding sequences of *WsCYP98A* and *WsCYP76A* cDNAs were expressed in *Escherichia coli* BL21 (DE3) using pGEX4T-2 expression vector. Quantitative real-time PCR analysis indicated that both genes express widely in leaves, stalks, roots, flowers and berries with higher expression levels of *WsCYP98A* in stalks while *WsCYP76A* transcript levels were more obvious in roots. Further, transcript profiling after methyl jasmonate, salicylic acid, and gibberellic acid elicitations displayed differential transcriptional regulation of *WsCYP98A* and *WsCYP76A*. Copious transcript levels of both P450s correlated positively with the higher production of withanolides.

**Conclusions:**

Two A-types P450 *WsCYP98A* and *WsCYP76A* were isolated, sequenced and heterologously expressed in *E. coli*. Both P450s are spatially regulated at transcript level showing differential tissue specificity. Exogenous elicitors acted as both positive and negative regulators of mRNA transcripts. Methyl jasmonate and salicylic acid resulted in copious expression of *WsCYP98A* and *WsCYP76A*. Enhanced mRNA levels also corroborated well with the increased accumulation of withanolides in response to elicitations. The empirical findings suggest that elicitors possibly incite defence or stress responses of the plant by triggering higher accumulation of withanolides.

**Electronic supplementary material:**

The online version of this article (doi:10.1186/s12896-014-0089-5) contains supplementary material, which is available to authorized users.

## Background

Cytochrome P450s form a huge superfamily of heme-containing monooxygenases present in all domains of life. These are pivotal in detoxification of xenobiotics, drug metabolism, assimilation of carbon sources and formation of secondary metabolites. Presently, there are more than 18500 P450 genes that have been identified across all the kingdoms of life [1,2]. The preponderance of P450s is more in plants per species than in animals and their content in angiosperm genomes can reach up to 2% [3,4]. Plant cellular and metabolic processes are dependent on P450s to drive the reactions such as hydroxylation, oxidative demethylation, desaturation, epoxidation, oxidative rearrangement of the carbon skeleton and oxidative C–C bond cleavage [5]. P450s are endoplasmic reticulum (ER) localised, requiring auxiliary reductases for the activation of molecular oxygen for different reactions. These reductases transfer two electrons in a single step from cofactor NAD(P)H to heme centre of P450s [6]. P450 monooxygenases constitute a highly regio and stereo-specific class of definite substrate-specific enzymes. By gene annotations approximately, 1% of the total genes in *Arabidopsis thaliana* and other model plants are found to be cytochrome P450s. *Arabidopsis* genome contains 244 genes and 28 pseudo-genes representing cytochrome P450 [3]. Involvement of P450s in diverse physiological processes *in vivo,* leads to biosynthesis and catabolism of huge array of complex metabolites like lignin, pigments, defence compounds, fatty acids, hormones and signaling molecules [7–9].

Owing to their regio and stereo-specific catalysing versatility, these are potential targets for industrial biocatalysis. P450s have been applied in industry for the investigation of new drugs, medicine or xenobiotics [10–12]. Remarkable variety of chemical reactions catalysed and enormous number of substrates attacked, have earned P450s the reputation of “the most versatile biological catalysts in nature” [13–15]. Thus, emphasizing identification and characterization of P450s for elucidation of various biosynthetic pathways. Plant P450s are presumed to be polyphyletic in their origin and generally classified into two main clades, A-type and non-A-type. A-type P450s are involved in ‘plant specific metabolism’ and are responsible for the biosynthesis of diverse natural products [7].

*Withania somnifera* is a medicinal plant of immense repute known to synthesize a suite of low molecular weight natural products such as flavonoids, alkaloids, terpenoids, tannins, resins and sterols through secondary metabolism [16–18]. These metabolites are derived from distinct metabolic pathways. The main constituents reported for significant pharmacological activities are withanolides, a group of naturally occurring triterpenoids. Withanolides are synthesized *via* both mevalonate (MVA) and non-mevalonate (MEP/DOXP) pathways [19]. Recently, we have elucidated the regulatory role of three branch point oxidosqualene cyclases (β-amyrin synthase, lupeol synthase and cycloartenol synthase) which showed that repression of β-amyrin synthase and lupeol synthase led to diversion of common precursor flux towards cycloartenol synthase resulting in enhanced accumulation of withanolides [20]. We have also characterized two paralogs of cytochrome P450 reductase (*WsCPR1* and *WsCPR2*) wherein *WsCPR2* expression was inducible in response to exogenous elicitors [21]. Two more genes namely squalene synthase and squalene epoxidase were also characterized from same plant species [22]. The regulation pattern of squalene synthase, squalene epoxidase, cycloartenol synthase and *WsCPR1* and *WsCPR2* in leaves and roots at various developmental stages in relation to dynamics of withanolides accumulation, indicated towards their tissue-specific biosynthesis [22]. In our earlier findings, we have reported that exogenous application of methyl jasmonate (MeJA) and salicylic acid (SA) increases withanolide content due to the presence of stress regulatory elements within the upstream promoter regions of their genes [20,23].

We, in present study report isolation, heterologous expression and transcript profiling of two A-type P450 monooxygenases viz. *WsCYP98A* (GenBank: HM585369) and *WsCYP76A* (GenBank: KC008573). These are supposed to be involved mainly in the shikmate and phenylpropanoid pathway biosynthesizing the various defence molecules during biotic and abiotic stress. CYP98A (p-coumarate 3-hydroxylase) catalyzes the meta-hydroxylation of *p*-coumarate derivatives for the biosynthesis of lignins and chlorogenic acid, which constitutes a crucial bottleneck in phenylpropanoid pathway [24]. The members of CYP76 gene family characterized from many plant species are known to catalyse various oxidative reactions. For instance, *Catharanthus roseus* CYP76A26 converts both iridodial and iridotrial into 7-deoxyloganetic acid. Whereas, *C. roseus* CYP76B6 and CYP76C1 from *A. thaliana* execute hydroxylation of geraniol [25,26].

In present investigation, full length genes of *WsCYP98A* and *WsCYP76A* were cloned and heterologously expressed in *Escherichia coli. WsCYP98A* shares maximum homology with reported *CYP98A* of *Capsicum annum* (95%) and *Solanum tuberosum* (94%) whereas, *WsCYP76A* was similar to *CYP76A2* of *S. tuberosum* (83%) and *S. melongena* (83%). The phylogenetic analysis demonstrated that both genes belong to their respective families and are close homologs to other *CYP98A* and *CYP76A* genes of Solanaceae family. The prediction of three dimensional structures, ligand binding site and conserved amino acid residues of both proteins were done using homology modelling analysis. The transcript profiling of *WsCYP98A* and *WsCYP76A* in different tissues of *W. somnifera* was performed using quantitative real time PCR (qRT-PCR). Expression profiles in response to exogenous elicitors namely MeJA, SA and gibberellic acid (GA_3_) were also studied for both the P450s. Empirical findings reveal their possible role in defence mechanism *via* enhanced accumulation of withanolides. Purview of literature suggests that this is the only report of type-A *WsCYP98A* and *WsCYP76A* from *W. Somnifera*.

## Methods

### Plant material

A WS-3 rich genetic stock of *W. somnifera* designated as WS-Y-08 grown at IIIM experimental farm (CSIR-Indian Institute of Integrative Medicine, Jammu, India, 32°44′N longitude, 74°55′E latitude; 305 m in altitude) was used as a source material. *In vitro* plants were raised through induction of axillary buds. These cultures were used for MeJA, SA and GA_3_ treatments. Samples were collected, frozen immediately in liquid nitrogen, and stored at −80°C.

### RNA isolation and cDNA synthesis

Total RNA was extracted using Trizol reagent (Sigma, St. Louis, USA) as per manufacturer’s protocol. Concentration of isolated RNAs was estimated by measuring the absorbance at 260 nm in a spectrophotometer (AstraAuriga, Cambridge, UK). Further, quality of RNA was assessed by determining the ratio of absorbance at 260 and 280 nm (*A*_260/280_) and formaldehyde-denatured agarose gel electrophoresis. Total RNA (5 μg) was incubated with RNase free DNase (Fermentas, Burlington, Canada) at 37°C for 30 min. First strand cDNA synthesized using the RevertAid cDNA synthesis kit (Fermentas, Burlington, Canada) in a total volume of 20 μl containing 3 μg total RNA, 10 mM dNTPs, 10 μM oligo (dT) primer, 1 μl M-MuLV reverse transcriptase (200 U/μl) and 1X first strand buffer (250 mM Tris–HCl, pH 8.3, 250 mM KCl, 20 mM MgCl_2_, 50 mM DTT). The reaction was incubated for 60 min at 42°C followed by 5 min at 70°C to inactivate the reverse transcriptase.

### Amplification of *WsCYP98A* and *WsCYP76A*

Degenerate primers (Table 1) based on the conserved regions of P450s were designed by multiple sequence alignment of different P450 monooxygenases sequences retrieved from the GenBank database at National Center for Biotechnology Information (NCBI). Optimization of polymerase chain reaction conditions allowed amplification of cDNA fragments of *WsCYP98A* and *WsCYP76A* under following cycling conditions: one cycle of 94°C for 3 min, 35 cycles of 94°C for 30 s, 55–60°C for 30 sec and 72°C for 1 min followed by a final extension of 72°C for 10 min in a thermal cycler (Bio-Rad Laboratories, Hercules, USA). Amplicons examined by agarose gel electrophoresis were cloned into pTZ57R/T vector (Fermentas, Burlington, Canada), and then transformed into *E. coli* DH5α host strain. Cloned amplicons were sequenced using an automated DNA sequencer (ABI Prism 3130XL; Applied Biosystems, Foster City, USA). The nucleotide sequences obtained were analysed using the similarity search BLAST program and subsequently used for designing gene specific primers (GSPs).

**Table 1:** **List of primers used in the study**

**Primers**	**Sequence (5′ − 3′)**	**Direction**
**Degenerate**		
CYP450-1 F.	CCN(A/C/G/T)TAY(C/T)GGH(A/C/T)GD(G/A/T)TACTGGAGR(A/G)CAG	Forward
CYP450-1R.	AGTCD(G/A/T)GD(G/A/T)CAW(A/T)D(G/A/T)GCCCATTCD(G/A/T)AG	Reverse
CYP450-2 F.	ATATGGGCY(C/T)GATTATGGV(G/A/C)CCTCA	Forward
CYP450-2R.	AGCATH(A/C/T)AGH(A/C/T)GGAGTTGGAGGY(C/T)TG	Reverse
**5′ and 3′ RACE**		
5′ Adapter*	GCUGAUGGCGAUGAAUGAACACUGCGUUUGCUGGCUUUGAUGAAA	
5′ RACE-OUT*	GCTGATGGCGATGAATGAACACTG	Forward
5′ RACE-IN*	CGCGGATCCGAACACTGCGTTTGCTGGCTTTGATG	Forward
5′ CYP98-OUT	TGTTATCAGAATTATTGCAATCTCTGT	Reverse
5′ CYP98-IN	AGCCGTAACCTCATCTTCACGAATGG	Reverse
5′ CYP76-OUT	CGTCTCTGTACCAGCTAAAAACATTTCCAC	Reverse
5′ CYP76-IN	GATCTCATGTTCTGATAACTTGGCTGGTT	Reverse
3′ Adapter*	GCGAGCACAGAATTAATACGACTCACTATAGGT_12_V(G/A/C)N(A/C/T/G)	
3′ RACE-OUT*	GCGAGCACAGAATTAATACGACT	Reverse
3′ RACE- IN*	CGCGGATCCGAATTAATACGACTCACTATAGG	Reverse
3′ CYP98-OUT	TGATCGGCTATGAGCGCGTGATGAA	Forward
3′ CYP98-IN	AAGCGCTAAGGTTGCAGCCTCCAA	Forward
3′ CYP76-OUT	AGAGATCCTGAATGTTGGGATGACCCTAT	Forward
3′ CYP76-IN	AGAGATCCTGAATGTTGGGATGACCCTAT	Forward
**Full length**		
FullCYP98F	ATGGCAATTCCCTTAGCTGCTGCAATCCCTC	Forward
FullCYP98R	TTATATGTCCACTGCAATTCGTTTATACAACTCAGC	Reverse
FullCYP76F	ATGGAATGGGAATGGAGCTATTTGT	Forward
FullCYP76R	CTAGATAAGATTGATGAGTGTCTCA	Reverse
**Expression (** ***E. coli*** **)**		
CYP98*BamHI*F	CG**GGATCC**ATGGCAATTCCCTTAGCTGCTGCAA	Forward
CYP98*SalI*R	GCTC**GTCGAC**TTATATGTCCACTGCAATTCGT	Reverse
CYP76*BamHI*F	CG**GGATCC**ATGGAATGGGAATGGAGCTATTTGT	Forward
CYP76*SalI*R	GCTC**GTCGAC**CTAGATAAGATTGATGAGTGTCTCA	Reverse
**Real-Time analysis**		
RTCYP98F	GTGATGGATGAACAAGGAAACGA	Forward
RTCYP98R	CCGTGCTTAGAAAATGCATCCTC	Reverse
RTCYP76 F.P	TAGAATGGGCACTAGCAGAGCTTTTGCG	Forward
RTCYP76 R.P	CTTGCATATAAGGGAGATTGTCGATGTCGTT	Reverse
ActinF	GAGAGTTTTGATGTCCCTGCCATG	Forward
ActinR	CAACGTCGCATTTCATGATGGAGT	Reverse

*Primers marked with star were provided with the kits. Added restriction sites for cloning into pGEX4T-2 vector are bold.

### 5′ and 3′ RACE PCR

Remaining 5 & 3 cDNA ends of putative *WsCYP98A* and *WsCYP76A* were obtained using RLM-RACE kit according to the product manual (Ambion, Austin, TX, USA). cDNAs obtained were subjected to nested PCR using GSPs as listed in Table 1. Two rounds of PCR were performed, first round with 5′ RACE-OUT corresponding to the 5′ RACE adapter sequence and 5′ CYP98-OUT and 5′ CYP76-OUT as specific outer primers, followed by a second round of PCR with 5′ RACE-IN as inner adapter specific primer and 5′ CYP98-IN and 5′ CYP76-IN as inner GSPs. In both rounds, PCR reactions of 50 μl containing 1.0 μl cDNA as template (except for second round where amplified products of 1st round were used as template), 2 μl of 10 μM 5′ CYP98-OUT and 5′ CYPP76-OUT , 2 μl of 5′ RACE-OUT, 45.0 μl master Mix (34.5 μl PCR-grade water, 10 mM Tris HCl; pH 9.0, 50 mM KCl, 2.5 mM MgCl_2_, 200 μM dNTPs, 2.5 U Taq DNA polymerase) were subjected to following cycling conditions: One cycle of 94°C for 3 min and 35 cycles of 94°C for 30 s, 60°C for 30 s, 72°C for 2 min with a final step at 72°C of 10 min. Similarly for 3′ RACE, first strand cDNA was synthesized from total RNA using supplied 3′ RACE adapter primer. cDNA was subjected to nested PCR using outer and inner primers specific to 3′ RACE adapter along with 3′ GSPs with same reaction volume and cycling conditions as described for 5′ RACE. The nested amplification products of both 5′ RACE PCR and 3′ RACE PCR were purified and cloned into pTZ57R/T cloning vector (Fermentas, Burlington, Canada). The ligation mixtures were transformed into *E. coli* cells (New England Biolabs, Herts, UK). The clones were picked individually and amplified in 10 mL of Luria-Bertani (LB) medium at 37°C overnight. The plasmid DNA from each clone was extracted using a DNA plasmid Miniprep Kit (Promega, Madison, USA) and sequenced using M13 primers.

### Full-length cloning of *WsCYP98A* and *WsCYP76A*

By comparing and aligning the sequences of core fragments, 5′ RACE and 3′ RACE products, the full-length cDNAs of *WsCYP98A* and *WsCYP76A* were generated and subsequently amplified with primers FullCYP98F, FullCYP98R and FullCYP76F, FullCYP76R (Table 1). A high fidelity proof-reading DNA polymerase (New England Biolabs, Herts, UK) was employed for amplification under PCR conditions; One cycle of 94°C for 3 min , 35 cycles of 94°C for 30 s, 60°C for 30 s, 72°C for 1 min. The final extension was at 72°C for 10 min. PCR products were analysed on 1.2% agarose gels and visualized under UV light. The resulted amplified products were ligated in pJET vector and transformed into *E.coli* DH5α.

### *In silico* analysis

BLAST (http://blast.ncbi.nlm.nih.gov/Blast.cgi) was used to find similarity of amplified P450s in GenBank database. Translate tool (http://www.expasy.ch/tools/dna.html) was used to predict the open reading frame (ORF). The properties of deduced amino acid sequences of *WsCYP98A* and *WsCYP76A* were estimated by using ProtParam (http://www.expasy.ch/tools/protparam.html), SPLIT v.4.0 (http://split.pmfst.hr/split/4/) and TMHMM (http://www.cbs.dtu.dk/services/TMHMM/) programs. Protein sequences were retrieved from the GenBank through BLASTp algorithm at the National Center for Biotechnology Information (NCBI) using *WsCYP98A* and *WsCYP76A* sequences as query and several homologous sequences of different plant species were selected. Multiple sequence alignment was performed using ClustalX program with default parameters [27]. For phylogenetic analysis, sequences were aligned employing ClustalW program (http://www.ebi.ac.uk/Tools/msa/clustalw2/) and phylogenetic tree was constructed by Neighbor-joining method using MEGA 6 software [28]. Bootstrap analysis with 100 replicates was also conducted in order to obtain confidence levels for the branches. Protein secondary structures were determined by SOPMA program [29].

### Prediction of three-dimensional structures of *WsCYP98A* and *WsCYP76A*

Three-dimensional structures of *WsCYP98A* and *WsCYP76A* were predicted using Phyre^2^ server (Protein Homology/analogY Recognition Engine V 2.0) [30] using crystal structure of cytochrome P450 1a2 (PDB: 2hi4) as a template. Ligand binding sites were predicted using 3DLigandSite. Ramachandran plot analysis of both *WsCYP98A* and *WsCYP76A* was performed online using RAMPAGE web server [31]. Structurally, evolutionary and functionally important regions were identified in deduced protein sequences by ConSurf [32].

### Recombinant protein expression in *E. coli*

Full length coding sequences of *WsCYP98A* and *WsCYP76A* genes were modified by adding restriction sites (Table 1). Full length primers were modified by adding *BamH*I*/Sal*I restriction sites for both genes for directional cloning in pGEX4T-2 vector and heterologous expression in *E. coli*. ORFs containing modified restriction sites were excised from pJET cloning vector and ligated into pre-digested, purified pGEX4T-2. Further, ligated mixtures containing pGEX-*WsCYP98A* and pGEX-*WsCYP76A* expression cassettes were transformed into *E. coli* BL21 (DE3). Individual positive colony harbouring transformed expression cassettes were inoculated separately into 100 ml of LB containing 100 μg/ml of ampicillin and incubated overnight at 37°C. 1% culture was transferred into 100 ml of LB media containing the corresponding antibiotic and incubated at 37°C, until optical density (*A*_600 nm_) reached 0.4-0.5. Protein expression was induced by adding Iso-propyl β-D-1-thiogalactopyranoside (IPTG; Fermentas, Berligton, Canada) into the cultures at the concentration of 0.2 mM to 1 mM. The cultures were constantly incubated at 25°C for 8 h. The induced bacterial cells were harvested at an interval of 2 h by centrifugation and resuspended in 6X sodium dodecyl sulphate–polyacrylamide gel electrophoresis sample buffer (SDS-PAGE; 0.375 M Tris pH 6.8, 12% SDS, 60% glycerol, 0.6 M DTT, 0.06% bromophenol blue). The expression of target proteins was analysed on 10% SDS-PAGE.

### Tissue-specific gene expression analysis using qRT-PCR

To study tissue-specific expression, total RNA was extracted from leaves, stalks, roots, flowers and berries respectively. Total RNA (5 μg) was incubated with RNase free DNase (Fermentas, Burlington, Canada) at 37°C for 30 min. cDNA was synthesized from 3 μg of RNA using RevertAid cDNA synthesis kit according to manufacturer’s instruction. qRT-PCR amplification was performed using the Step One Real-time PCR System (Applied Biosystems, Foster City, USA). Briefly, the standard 20 μl of reaction included 0.2 μL cDNA template, 200 nM of each primers, and 10 μL SYBR Premix Ex **(**Takara, Otsu, Japan**)** under following cycling conditions: one cycle of 94°C for 1 min, 40 cycle of 94°C for 10 s, 60°C for 15 s and 72°C for 15 s. Two primers, Actin F and Actin R were used to amplify housekeeping actin gene as control. All samples were analysed in triplicate and the specificity of each primer pair was validated by a dissociation curve (a single peak was observed for each primer pair). The real-time PCR amplification data were exported into Microsoft Excel and gene expression levels were calculated based on the comparative C_t_ method [33].

### Effect of elicitors on the expression of *WSCYP98A* and *WsCYP76A*

For elicitor treatment the micro-propagated plantlets were pre-cultured in Murashige and Skoog (MS) liquid medium supplemented with 3% sucrose, inositol (100 mg l^−1^) and incubated at 25 ± 1°C under 16 h photoperiod with light intensity of 30 μmol m^−2^ s^−1^ provided by cool, white fluorescent tubes of 40 W (Philips, Calcutta, India) for 2 weeks. Relative humidity was maintained at 50-60%. The plantlets growing in liquid MS medium were supplemented with MeJA (0.1 mM), SA (0.1 mM), and GA_3_ (0.1 mM) dissolved in ethanol and untreated were kept as control with same amount of ethanol. The tissue from each treated sample was harvested after 6, 12, 24 and 48 h for RNA isolation. cDNA was obtained from each treated sample including control, using the same RNA isolation and cDNA synthesis protocols as described above. Effect of elicitor treatment on expression profile was studied using qRT- PCR as described above.

### Withanolide analysis using HPLC

Withanolides were extracted and quantified as described by Dhar *et al*. [34]. Concisely, samples of *W. somnifera* were powdered and extracted with ethanol-water (50:50; *v/v*) with magnetic stirring at room temperature (25 ± 2°C). Extracts were filtered and the solvent was removed under vacuum. The extracts (20 mg/ml) obtained from each sample of the plant material were prepared in HPLC-grade methanol–water (50:50; *v/v*) for quantitative analysis. 1.2 mg per 2 ml of the standards of withanolide A (WS-1), withanone (WS-2) and withaferine A (WS-3) were prepared in HPLC-grade methanol. HPLC analysis was performed with Shimadzu HPLC system (Shimadzu, Tokyo, Japan) equipped with 515 quaternary gradient pump, 717 Rheodyne injector, 2996 PDA detector and CLASS-VP software v 6.14. All samples were filtered through 0.45 μM filters (Millipore, Bedford, USA). Extracts of *W. somnifera* samples were separated on a RP-18e (4.6 × 100 mm, 5 μm) (Merck, Bangalore, India) column. The mobile phase consisted of methanol–water (60:40; *v/v*) delivered at a flow rate of 0.5 ml/min. The samples were analyzed at 30°C to provide efficiency to the peaks. The UV chromatograms were recorded at 237 nm.

## Results and discussion

### Molecular cloning of *WsCYP98A* and *WsCYP76A*

P450 superfamily contain genes of diverse functions involved both in primary as well as secondary metabolism. These are heme-thiolate proteins containing iron atom coordinated to a proximal cysteine and receive electrons from NAD(P)H *via* a FAD-domain of auxiliary reductases. In the present investigation, two monooxygenases from *W. somnifera, WsCYP98A* and *WsCYP76A* have been isolated and expressed in *E. coli* BL21 (DE3). Quantitative assessment of gene expression pattern in different plant organs and relative transcript abundance in response to exogenous elicitors was also evaluated and corroborated with withanolide production to extrapolate and infer their possible role during biotic and abiotic stresses.

**Figure 1:**
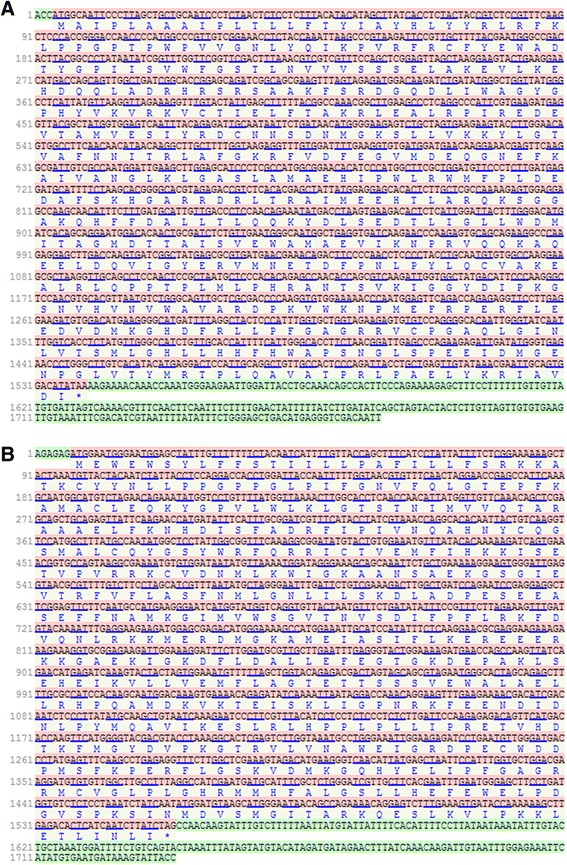
**Nucleotide and the deduced amino acid sequence of** ***WsCYP98A*** **(A) and** ***WsCYP76A*** **(B) from** ***Withania somnifera*** **.** The start codon (ATG) present at 4th and 7th positions whereas stop codons at 1552 and 1537 bp, respectively.

Full length cDNAs of *WsCYP98A* and *WsCYP76A* genes were obtained from the leaf tissue of WS-3 rich chemo-variant by degenerate PCR and RACE methods (*WsCYP98A*: HM585369 and *WsCYP76A*: KC008573). Degenerate primers based on conserved regions were designed by employing multiple sequence alignment strategy using ClustalW. A 550 bp and 520 bp core fragments were obtained from initial RT-PCR reactions. The amplicons were confirmed as members of CYP98 and CYP76 families by sequencing and similarity searches using BLASTn. Further, extension of both amplified core fragments toward 5′ and 3′ ends by using RACE-PCR succeeded in amplification of remaining cDNA portions. Using GSPs designed from start codons and stop codons, 1536 bp and 1548 bp open reading frames (ORFs) were amplified from the pool of leaf cDNA designated as *WsCYP98A* and *WsCYP76A* and theses encoded 511 and 515 amino acid residues respectively (Figure 1A and B). The first methionine as per First-AUG rule was considered as initiator codon. *WsCYP98A* contains downstream untranslated region (UTR; 230 bp) at 3′ ends whereas *WsCYP76A* comprises of 3′-UTR of 180 bp. Similarity search showed *Ws*CYP98A shares 65-95% homology with protein sequences of many other species while *Ws*CYP76A shares 39-89% homology.

### *In silico* characterization of deduced *Ws*CYP98A and *Ws*CYP76A

**Figure 2:**
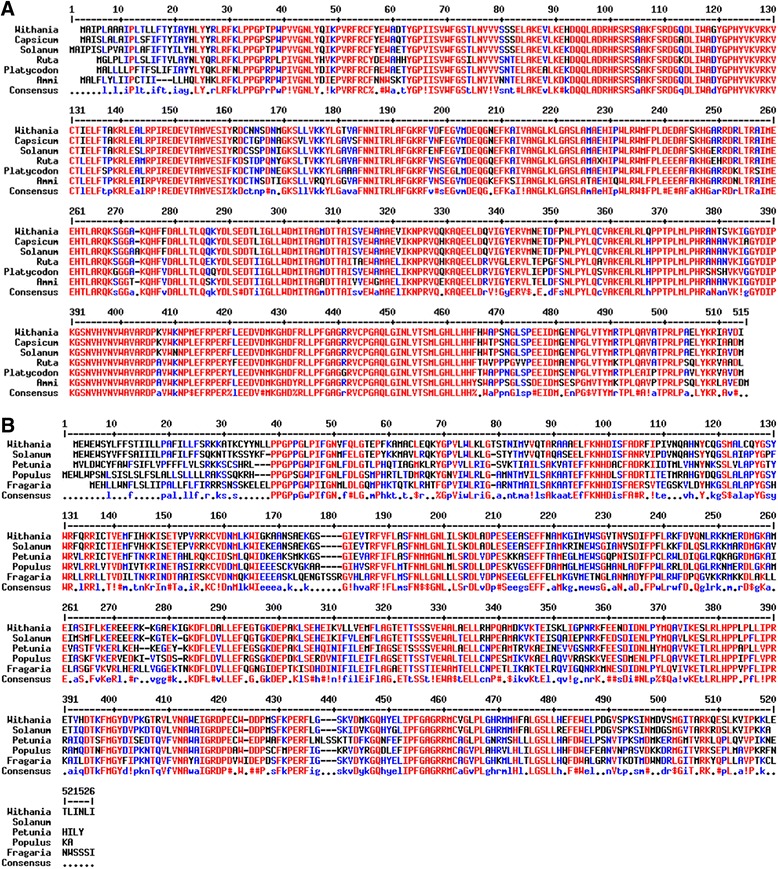
**Multiple sequence alignment of deduced amino acid sequences of** ***WsCYP98A*** **and** ***Ws*** **CYP76Awith their respective homologs using Multalign tool.** For *Ws*CYP98A **(A)**, sequences used for alignment were from *Withania somnifera* (*WsCYP98A*: HM036710), *Solanum lycopersicum* (ACF17644.1) *Capsicum annuum* (ACF17644.1)*, Ruta graveolens (AEG19446.1), Platycodon grandiflorus* (AEM63674.1), *Ammi majus* (AAT06912.1). Consensus residues present were shown below. For *Ws*CYP76A **(B)**, sequences used for alignment were from *Withania somnifera* (*WsCYP98A*: HM036710), *Solanum lycopersicum* (XP_004249463), *Populus trichocarpa* (XP_002309107.1), *Fragaria vesca* (XP_004305369.1), *Petunia x hybrida* (BAC53892.1). Consensus residues present were shown below.

**Figure 3:**
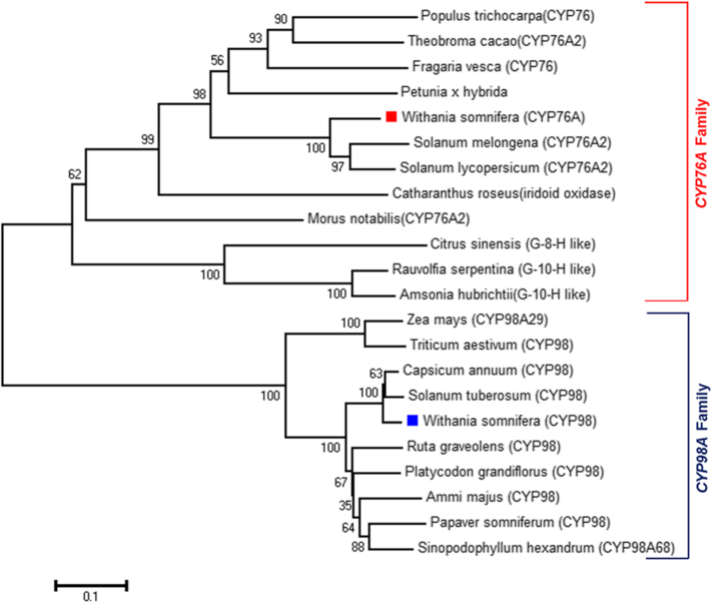
**Phylogenetic analysis of deduced amino acid sequences of** ***WsCYP98A*** **and** ***WsCYP76A*** **was inferred using the Neighbour-joining method employing MEGA 6.0 software.** For *WsCYP98A* total of 10 sequences and for *WsCYP76A*, 12 sequences including *Withania somnifera* were used for analysis. The sequences used were from following plant specieis: *Withania somnifera* (*WsCYP98A*: HM036710; *WsCYP76A*: GU808569), *Solanum lycopersicum* (ACF17644.1) *Capsicum annuum* (ACF17644.1) *Solanum tuberosum (XP_006347680.1), Ruta graveolens (AEG19446.1), Platycodon grandiflorus* (AEM63674.1), *Ammi majus* (AAT06912.1)*, Zea mays* (ACG25686.1), *Triticum aestivum* (CAE47490.1), *Papaver somniferum* (AFK73720.1), *Sinopodophyllum hexandrum* (AGC29945.1), *Solanum melongena* (CAA50648.1), *Solanumlycopersicum* (XP_004249463), *Populus trichocarpa* (XP_002309107.1), *Fragaria vesca* (XP_004305369.1), *Petunia x hybrida* (BAC53892.1), *Theobroma cacao* (XP_007027589.1), *Catharanthus roseus (iridoid oxidase;* AHK60833.1*)*, *Morus notabilis* (EXC33897.1), *Rauvolfia serpentina* (G-10-H like; AGX93053.1), *Amsonia hubrichtii* (G-10-H like; AGX93052.1, *Citrus sinensis* (G-8-H like; XP_006477331.1). Both P450 confined to separated clan and exhibited maximum similarity with their respective members of CYP98 and CYP76 families.

**Figure 4:**
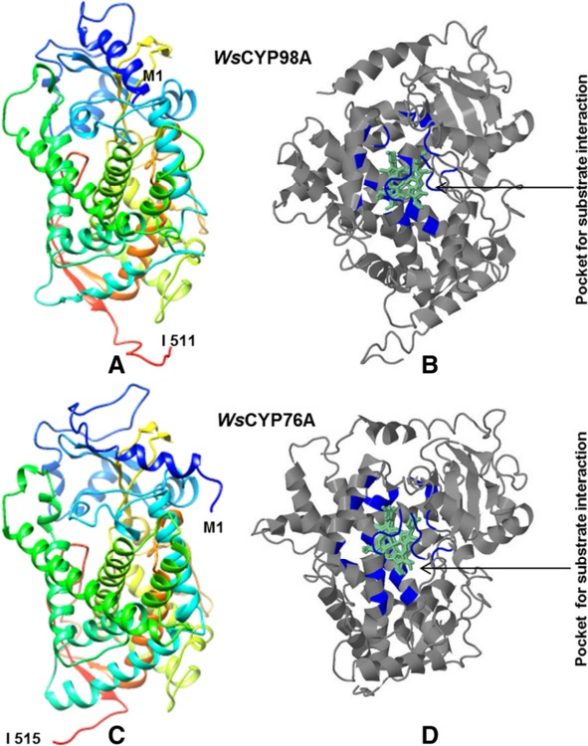
**Prediction of three dimensional structures of** ***Ws*** **CYP98A and** ***Ws*** **CYP76A using Phyre** ^**2**^ **based on homology modelling approach. A** & **C**: Cartoon display of the 3-D structures of *Ws*CYP98A and *Ws*CYP76A as predicted by Phyre^2^ using crystal structure of cytochrome P450 1a2 (PDB: 2HI4) as template.** B** & **D**: Predicted ligand (shown in green) binding sites as predicted by 3DLigandSite Web Server. The residues involved in substrate binding and active site are shown in the center core of structure.

*Ws*CYP98A and *Ws*CYP76A have predicted isoelectric points 8.13 and 7.99 and molecular masses of 58.0 kDa and 58.7 kDa respectively. Multiple sequence alignment with P450s of taxonomically diverse species showed presence of cysteine heme ligand signature which is characteristic signature of P450 sequences This motif was detected at positions 434–443 aa (FGAGRRVCPG) and 444–453 aa (FGAGRRMCVG) for *Ws*CYP98A and *Ws*CYP76A respectively (Figure 2A and B). Like all eukaryotic monooxygenases, a string of amino acid residues which helps in anchoring to ER membrane was detected at the N-terminal end of each monooxygenase. This membrane anchor region is also essential for normal interaction between P450s and their redox partners. Their presence was also predicted by TMHMM and SPLIT 4.0 bioinformatics programs (Additional file 1: Figure S1). To ascertain the degree of evolutionary relatedness, Neighbor-joining phylogenetic tree was constructed with MEGA 6.0 software from the ClustalW alignment of *Ws*CYP98A and *Ws*CYP76A with a number of homologous P450 sequences of different plants retrieved from the NCBI GenBank database. *Ws*CYP98A and *Ws*CYP76A corresponded to two separate phylogenetic clans in accordance with the amino acid similarity among their proteins (Figure 3). Prediction of secondary structure of *Ws*CYP98A and *Ws*CYP76A proteins by SOPMA program revealed that they consist of α-helixes (52.84%; 50.68%), β-turns (6.07%; 4.47%) joined by extended strands (8.61%; 10.49%), and random coils (32.49%; 34.37%). Three dimensional structural models for *Ws*CYP98A and *Ws*CYP76A were generated using Phyre^2^ with >90% accuracy on the basis of homology based modelling using cytochrome P450 1a2 (PDB: 2HI4) as template (Figure 4A and C). The amino acid residues involved in ligand binding were also predicted using the 3DLigandSite tool as depicted in Figure 4B and D. Analysis of the evolutionary conservation of *Ws*CYP98A and *Ws*CYP76A amino acids were performed using ConSurf program. Several residues with high scores were found to be functional and structural residues of the proteins by ConSeq servers (Additional file 2: Figure S2). Ramachandran plot analysis of *Ws*CYP98A showed 461 aa (90.6%) residues in the most favourable region, 32 aa (6.3%) residues in the additional allowed region and 16 (3.1%) in the outlier region. Similarly, in case of *Ws*CYP76A, 480 aa (93.6%) residues were in the most favourable region, 20 aa (3.9%) residues in the additional allowed region and 13 (2.5%) in the outlier region (Additional file 3: Figure S3).

### Heterologous expression in *E. coli*

**Figure 5:**
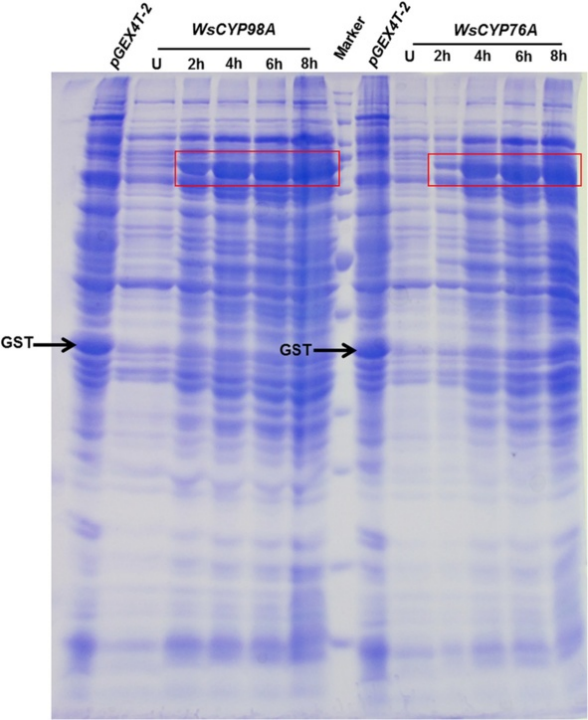
**Sodium dodecyl sulphate–polyacrylamide gel electrophoresis (SDS-PAGE: 10%) pattern of proteins obtained from** ***E.coli*** **BL21 (DE3) transformed with pGEX-** ***WsCYP98A*** **and pGEX-** ***WsCYP76A*** **.** Lane 1 & 8; Cell lysate of *E. coli* BL21 (DE3) cells containing the empty vector pGEX-4 T2 obtained at 4 h post-induction with 0.8 mM IPTG. Lane 2 & 9; Whole cell lysate of uninduced *Ws*CYP98A and *Ws*CYP76A, Lane 7; Standard protein marker Lane 3–6 & 9–12; Cell lysate of *Ws*CYP98A and *Ws*CYP76A induced with 0.8 mM IPTG harvested after 2 h, 4 h, 6 h and 8 h respectively.

For heterologous expression, entire coding sequences of *WsCYP98A* and *WsCYP76A* cDNAs were expressed in *E. coli* using pGEX 4 T-2 expression vector system. The ORFs were released from pJET-*WsCYP98A* and pJET-*WsCYP76A* using *BamH*I*/Sal*I restriction enzymes, and inserted into vector pGEX4T-2. The recombinant expression vectors with the inserted *WsCYP98A* and *WsCYP76A* constructs were identified by PCR analysis and restriction digestion using *BamH*I*/Sal*I. Heterologous expression of proteins was induced with different concentrations of IPTG. SDS-PAGE analysis demonstrated that optimum expression of proteins was observed at 25°C using 0.8 mM IPTG after 6–8 h of induction. The fusion protein having molecular weight of ~84.06 kDa and ~84.7 kDa appeared in the lysate of recombinant *E. coli* transformed with the expression cassettes pGEX-*WsCYP98A* and pGEX-*WsCYP76A*, respectively (Figure 5). The apparent molecular masses of these recombinant proteins were in good agreement with molecular mass of GST (26 kDa) and those calculated from the deduced primary structures of *Ws*CYP98A and *Ws*CYP76A proteins. Previously, we were able to optimize the functional expression of *WsCPRs* in soluble fraction with its membrane anchor [21]. Nevertheless, *Ws*CYP98A and *Ws*CYP76A were not expressed in soluble fraction in ample amount. Maximum amount of recombinant protein of two P450s were localized to inclusion bodies which hindered their purification.

### Tissue-specific gene expression analysis

**Figure 6:**
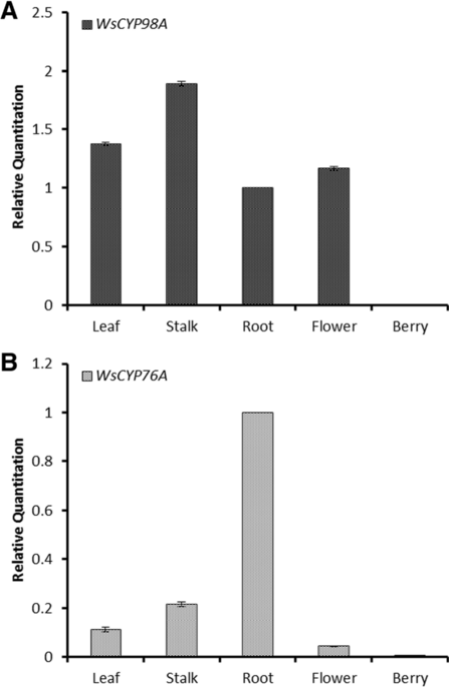
**Quantitative assessment of the expression of (A)** ***WsCYP98A*** **and (B)** ***WsCYP76A*** **in different tissues of** ***Withania somnifera.*** Data were compared and analysed with analysis of variance (*ANOVA*). Values are means, with standard errors indicated by bars, representing three independent biological samples, each with three technical replicates.

To study *WsCYP98A* and *WsCYP76A* gene expression pattern in different tissues of *W. somnifera*, cDNA libraries were prepared separately from RNA samples extracted from leaves, stalks, roots, flowers and berries (unripen) of four month old plant. Tissue-specific cDNAs were used as templates for qRT-PCR. The results showed highest expression of *WsCYP98A* in stalk (Figure 6A)*.* Similar findings have been reported for coumarate 3-hydroxylase in *A. thaliana* where the distribution of transcripts was more preponderant in stem. High frequencies of *CYP98A* transcripts have also been observed in poplar and pine xylem, soybean hypocotyl and stem, as well as cotton fibres indicating towards its higher expression in lignin synthesizing tissues [35–37]. However, two closely related homologs *CYP98A8* and *CYP98A9* expressed predominantly in floral tissues which is very distinct from other CYP98 members. The higher expression in floral tissue probably helps in pollen coat development [38]. *WsCYP76A* transcripts were found predominantly expressing in roots of *W. somnifera* (Figure 6B). Likewise, expression of *CYP76A3* was reported higher in petunia roots. Higher expression of *WsCYP76A* in roots possibly protects plant against attack by plant pathogens [39]. The comparative analysis of transcriptomes in *Salvia miltiorrhiza* showed abundant expression of *CYP76AH1* in roots rather than other plant tissues and found to be involved in tanshinone biosynthetic pathway [40]. The least expression of both monooxygenases was observed in berries.

### Effect of elicitors on *WsCYP98A* and *WsCYP76A*

*In vitro* cultures established *via* micro-propagation were used for MeJA, SA and GA_3_ treatments. Micro-shoots were grown for 2 weeks in MS liquid medium. Subsequently, micro-shoots were supplemented with MeJA (0.1 mM), SA (0. 1 mM) and GA_3_ (0.1 mM) for 48 h. The untreated were kept as control. Samples were harvested after 6, 12, 24, and 48 h of interval. Equal amounts of DNase-treated RNA (3 μg) of control and treated samples were converted into cDNA. The effect of MeJA, SA and GA_3_ on expression profile of *WsCYP98A* and *WsCYP76A* was studied using qRT-PCR.

**Figure 7:**
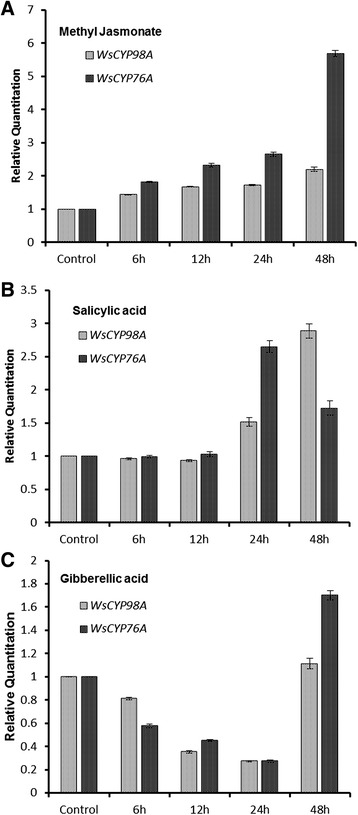
**Quantitative real-time analysis of** ***WsCYP98A*** **and** ***WsCYP76A*** **expression in micro-propagated** ***Withania somnifera*** **induced by (A) methyl jasmonate (MeJA; 0.1 mM), (B) salicylic acid (SA; 0.1 mM) and (C) gibberellic acid (GA**_**3**_ **; 0.1 mM) treatments. MeJA, SA and GA**_**3**_ **were added to the micro-propagated plantlets pre-cultured for 2 weeks on MS liquid medium.** The samples were harvested at time points of 6, 12, 24, and 48 h. Actin was kept as internal control. Data were compared and analysed with analysis of variance (*ANOVA*). Experiments were performed in triplicate with similar results with standard errors indicated by bars, representing three independent biological samples, each with three technical replicates.

One of the important endogenous plant hormones MeJA is known to modulate biosynthesis of many of the secondary metabolites that play crucial role in the adaptation of plants to various biotic and biotic stresses [41]. MeJA has been reported to stimulate the biosynthesis of alkaloids by inducing the expression of several genes from the monoterpenoid branch of the monoterpene indole alkaloid biosynthetic pathway [42]. In present study, MeJA induced expression of both P450s post 48 h of treatment. *WsCYP98A* showed 2-fold increase in expression after 48 h of application of MeJA while *WsCYP76A* showed 6-fold increase in the expression after 48 h of MeJA application (Figure 7A).

SA has been studied in detail as stress signaling molecule. It is implicated in signal transduction of numerous processes including the biosynthesis of some important secondary metabolites in plants [43–45]. SA has been demonstrated to induce the accumulation of triterpenoids, ginsenosides in *Panx ginseng* and glycyrrhizin in *Glycyrrhizaglabra*, respectively [44,46]. Production of sesquiterpenoids, such as bilobalide in *Ginkgo biloba* and artemisinin in *Artemisia annua* are also stimulated by exogenous application of SA [47,48]. SA treated plantlets showed upto 2.5-3 fold increase in accumulation of *WsCYP98A* and *WsCYP76A* after time points of 24 h and 48 h, respectively (Figure 7B). The results obtained are fully in agreement with the previous studies wherein microarray analysis revealed up-regulation of several P450s from *A. thaliana* in response to SA treatment [49]. Likewise, enhanced expression of *CYP98A5* gene has been noticed in *Physilus vulgaris* when leaves were treated with derivatives of salicylic acids [50]. The induction of *LjC3H* in *Lonicera japonium* has been induced by the treatment of MeJA and UV radiation [51,52]. Up-regulation of transcript levels of *WsCYP98A* and *WsCYP76A* in response to elicitors may be due to their possible implication in defence mechanism.

*CYP98A* is key gene of phenylpropanoid pathway which leads to formation of flavonoids, phenolic and lignin. During pathogens attack and wound healing, deposition of lignin and suberin synthesis occurs in the cell wall [53]. Thus, the abundance of transcripts of *WsCYP98A* in treated plantlets appears to be a defence response due to elicitor triggered stimulus. The expression of *WsCYP76A* have also enhanced in response to MeJA and SA treatment. Members of same family like *CYP76B6* is strongly induced by MeJA treatment along with other terpenoid indole alkaloid biosynthesis genes in *C. roseus* cell culture. After 48 h of MeJA treatment the expression of *WsCYP76A* was about 6-fold higher than control. Closely related homologs of CYP76 family are mainly involved in biosynthesis of phytoalexin and are mostly multifunctional oxidases such as CYP76M7, which seem to be involved in the production of antifungal phytocassanes [54]. *CYP76B6* has been shown to be a highly specialized multifunctional enzyme catalyzing two sequential oxidation steps leading to the formation of 8-oxogeraniol from geraniol [55].

GA_3_ is a diterpene plant growth regulator, controlling various growth and developmental process from seed germination to flower and fruit development. Broadly, GAs have been implicated in various cross talk mechanisms among different signaling pathways triggered by other plant growth regulators [56]. It impinges on a common transcription module of other phytohormones thus promoting many similar developmental responses in plants [57]. It has also been contended that GA_3_ orchestrates the metabolite fluxes between the primary and secondary metabolism influencing the production of isoprenoids and carotenoids [58,59]. Exogenous application of GA_3_ resulted in decrease in the expression of *WsCYP98A* as well as *WsCYP76A* (Figure 7C). Lowest transcript levels were observed after 24 h of treatment which indicated that GA_3_ acts as negative regulator of these two genes. After 48 h of treatment the transcript accumulation of *WsCYP98A* was comparable to control while as *WsCYP76A* showed 0.5-fold higher expression in comparison to control.

### Effect of elicitors on accumulation of withanolides

**Figure 8:**
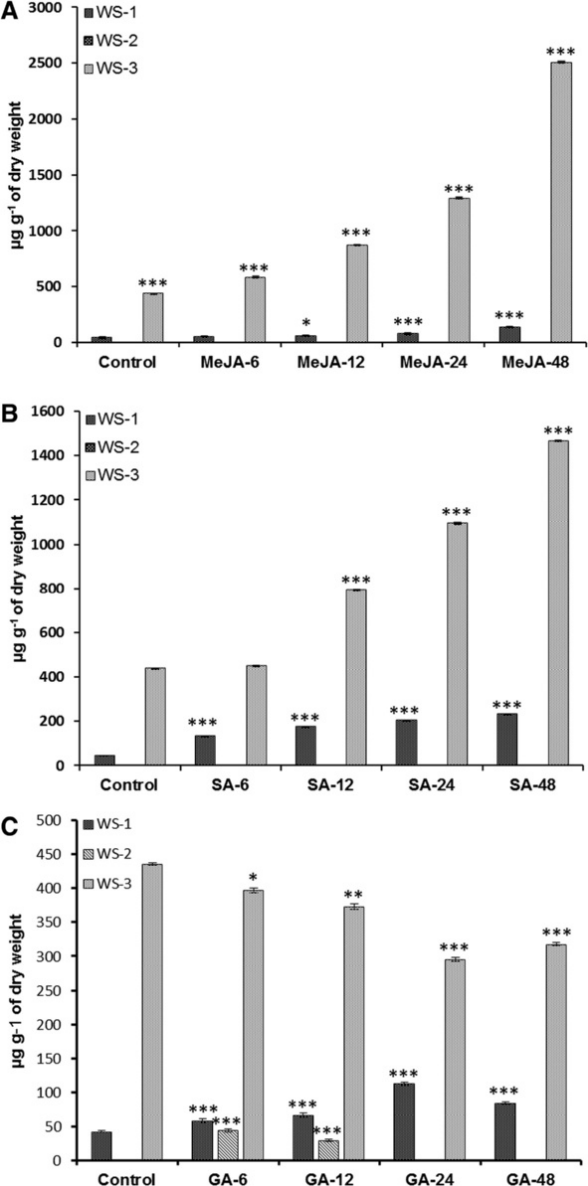
**Time-course effect of elicitor treatments on withanolides accumulation in response to (A) methyl jasmonate (0.1 mM), (B) salicylic acid (0.1 mM) and (C) gibberellic acid (0.1 mM) at different time points.** Variation in three key withanolides viz. withanolide A (WS-1), withanone (WS-2) and withaferine A (WS-3) was confirmed by HPLC analysis at 6, 12, 24 and 48 h. All values obtained were means of triplicate with standard errors. Time course accumulation of WS-1, WS-2 and WS-3 was statistically significant at *p < 0.05, **p < 0.01 and ***p < 0.001 levels.

Elicitor treatment is an efficient strategy to induce *de novo* synthesis of secondary metabolites. Endogenous signal molecules like MeJA and SA act as key signaling compounds in various stress responses and plants subjected to various vagaries often result in higher accumulation of secondary metabolites such as alkaloids, terpenoids, flavonoids, phenolic compounds and phytoalexins [60,61]. To establish a correlation between expression profiles and metabolite flux, withanolides extracted from the treated samples were subjected to HPLC analysis (Additional file 4: Figure S4). There was appreciable increase in WS-1 and WS-3 over a period of time in response to elicitor treatments. In MeJA treated samples, there was a significant increase in WS-1 (53.583 ± 1.02 − 137.530 ± 1.76 μg g^−1^ of dry weight) and WS-3 (584.012 ± 2.67 − 2504.937 ± 3.52 μg g^−1^ of dry weight) (Figure 8A). SA treated samples showed marked increase in both WS-1 (129.934 ± 1.04 − 230.864 ± 1.54 μg g^−1^ of dry weight) and WS-3(448.257 ± 2.59 − 1467.235 ± 2.58 μg g^−1^ of dry weight) with no traces of WS-2 (Figure 8B). Estimation of withanolide accumulation in GA_3_ treated samples demonstrated a gradual decline in WS-3 (397.326 ± 3.54 − 295.1673 ± 3.18 μg g^−1^ of dry weight) while as WS-1 increased moderately up to 24 h (58.475 ± 2.2 − 112.667 ± 2 μg g^−1^ of dry weight) but after 48 h there was a slight decrease in WS-1 (84.214 ± 2.01 μg g^−1^ of dry weight). WS-2 level also peaked at 6 h (44.323 ± 2.49 μg g^−1^ of dry weight) in GA_3_ treated plantlets followed by a decrease at 24 h (10.867 ± 0.73 μg g^−1^ of dry weight) and un-detectable concentration after 48 h (Figure 8C). Absence or low production of WS-2 is possibly linked to inherently lower levels of WS-2 accumulation even in cultivated accessions of *W. somnifera* [37]. Application of exogenously applied phytohormones has been shown to impinge on physiological and metabolic processes in many plant organs and cell cultures. In some of the reports accumulation of anthocyanins, glutathione and flavonoids have shown varied response to GA_3_ treatments [62].

In our previous reports, MeJA and SA up-regulated the expression of squalene synthase, squalene epoxidase and *WsCPR2* genes which play an important regulatory role in the phytosterol biosynthesis [63]. These elicitors are important components of signal transduction cascades activating plant’s defence response against pathogen attack and often result in increased metabolite accumulation.

## Conclusion

The increased demand of secondary metabolites for medicinal purposes coupled with low yield and supply has spurred the interest in enhanced production of bioactive metabolites. Recent developments and upsurge in synthetic biology approach in combination with rapid advances in systems biology and metabolic engineering have enabled the manipulation of genome of microorganisms to produce heterologous molecules in a manner that was previously much more demanding in terms of integrating the metabolic circuitries in heterologous host [64]. P450s play a critical role in heterologous and homologous systems as they are able to catalyse regio- and stereospecific hydroxylation reactions that are extremely difficult to carry out using chemical methods [65]. These P450-catalysed reactions are pivotal steps in the biosynthesis of variety of compounds and optimization of P450 enzyme activities are key targets in yield improvement efforts to render such approaches economically feasible [66]. The characterization of P450s and their role in biosynthesis of plant secondary metabolites is a fascinating area of investigation. Present endeavour was aimed towards identification and characterization of different P450 monooxygenases from *W. somnifera*. We were able to clone two important P450 monoxygenases from *Withania* which seem to play an active role in defence mechanism by coping up with biotic or abiotic stresses. It also gets reflected in higher accumulation of withanolides. The full length genes were successfully transformed in *E. coli* for heterologous expression. Time-course study revealed that IPTG had positive influence on protein expression in *E. coli*. Expression pattern for *WsCYP98A* and *WsCYP76A* in different plant tissues was studied using qRT-PCR which showed highest expression of *WsCYP98A* in stalk but the expression of *WsCYP76A* was more obvious in roots. In present study, MeJA and SA acted as positive regulators whereas GA_3_ behaved as a negative regulator for *WsCYP98A* and *WsCYP76A*. To establish a correlation between enhanced transcript levels in response to activation of defence mechanism we also analysed withanolide accumulation. As the elicitors play key role as signaling molecules during stress conditions and enhance production of secondary metabolites, we observed discernible changes in withanolide concentrations. MeJA elicitation significantly increased the WS-3 accumulation over a period of 48 h. These results were in conformity with our earlier studies where MeJA and SA mediated induction of squalene synthase, squalene epoxidase and *Ws*CPR2 also led to enhanced withanolide content [63,67]. GA_3_ was found to be a negative regulator and resulted in low levels of mRNA transcripts. The accumulation of withanolides in GA_3_ treated samples showed remarkable decrease in WS-3 and WS-1 accumulation. These results are positively corroborating with some of our earlier findings where the exogenous application of GA_3_ resulted in decrease in the cycloartenol synthase at translational level resulting in the decline of withanolide content [20]. Possibly, GA_3_ application diverts the flux towards the formation of primary metabolites for cell elongation and development because it has been reported to be associated with the promotion of germination, growth, and flowering [62]. For homologous modulation of withanolide biosynthetic pathway, we have successfully incited the induction of transformed hairy roots using *Agrobacterium rhizogenes*. Hairy roots provide an excellent experimental system to understand the regulatory role of different P450s with their redox partners for enhanced production of withanolides (Unpublished).

## Additional files

Additional file 1: Figure S1. Transmembrane domain prediction of **(A)** *WsCYP98A* and **(B)** *WsCYP76A* using TMHMM web server. Additional file 2: Figure S2. Evolutionary conserved key residue analysis of **(A)** *Ws*CYP98A and **(B)** *Ws*CYP76A identified using Consurf, an empirical Bayesian inference based web server. Residue conservation from variable to conserve is shown in blue (1) to violet (9). The residues involved in binding of the donor moieties are shown in the centre of the structures. Additional file 3: Figure S3. Ramachandran plot of *Ws*CYP98A *and Ws*CYP76A 3D models of *Withania somnifera* using RAMPAGE server. Most favoured regions are coloured red, additional allowed, generously allowed, and disallowed regions are indicated as yellow, light yellow and white fields, respectively. Additional file 4: Figure S4. HPLC analysis of withanolide A (WS-1), withanone (WS-2) and withaferin A (WS-3) at 6 h and 48 h from methyl jasmonate (0.1 mM), salicylic acid (0.1 mM) and gibberellic acid (0.1 mM ) treated micro-shoots of *Withania somnifera*.

## Abbreviations

ER: Endoplasmic reticulum
GSPs: Gene specific primers
RACE: Rapid amplification of cDNA ends
ORF: Open reading frame
UTR: Untranslated region
*E. coli*: *Escherichia coli*
IPTG: Isopropyl- β-D-thiogalactopyranoside
SDS-PAGE: Sodium dodecyl sulphate polyacrylamide gel electrophoresis
qRT-PCR: Quantitative real-time polymerase chain reaction
MeJA: Methyl jasmonate
SA: Salicylic acid
GA_3_: Gibberellic acid
HPLC: High performance liquid chromatography
