# Supplementary figures and images for: Molecular characterization of two A-type P450s, WsCYP98A and WsCYP76A from Withania somnifera (L.) Dunal: expression analysis and withanolide accumulation in response to exogenous elicitations
Source: BMC Biotechnol. 2014 Nov 23;14:89. doi: 10.1186/s12896-014-0089-5 (PMC4247701; doi:10.1186/s12896-014-0089-5)

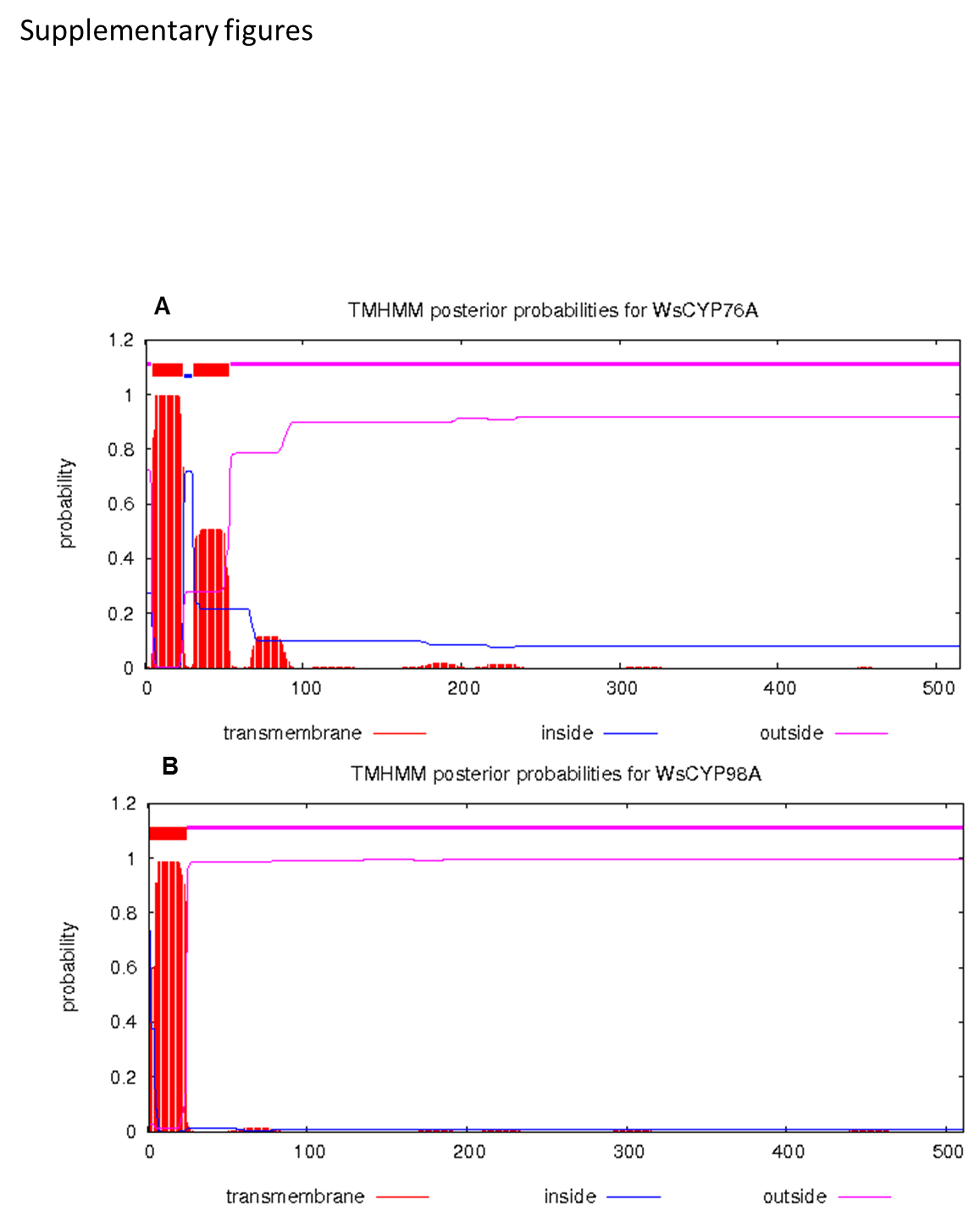

Supplement: Additional file 1: Figure S1. — Transmembrane domain prediction of (A) WsCYP98A and (B) WsCYP76A using TMHMM web server. [file 12896_2014_89_MOESM1_ESM.tiff]

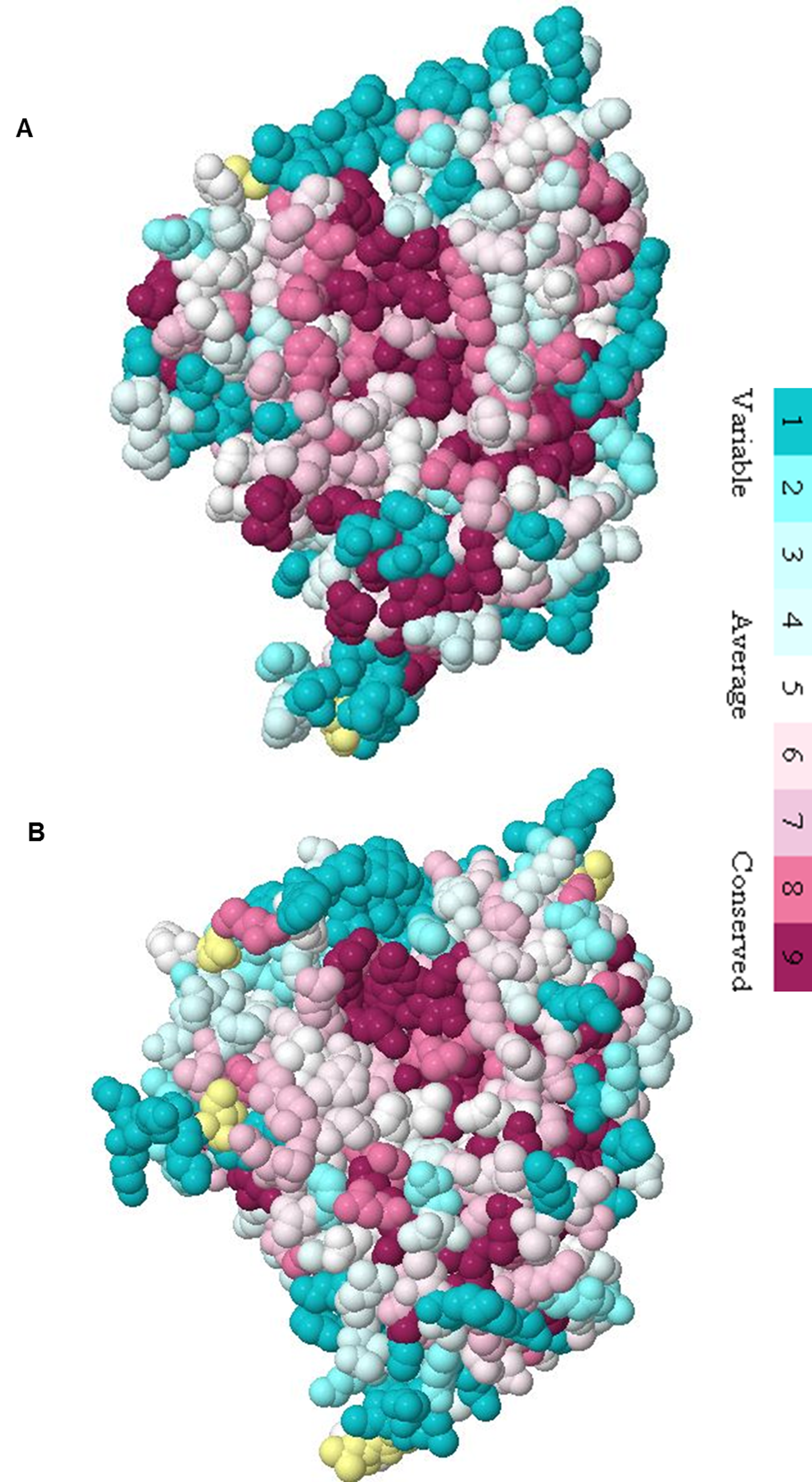

Supplement: Additional file 2: Figure S2. — Evolutionary conserved key residue analysis of (A) WsCYP98A and (B) WsCYP76A identified using Consurf, an empirical Bayesian inference based web server. Residue conservation from variable to conserve is shown in blue (1) to violet (9). The residues involved in binding of the donor moieties are shown in the centre of the structures. [file 12896_2014_89_MOESM2_ESM.tiff]

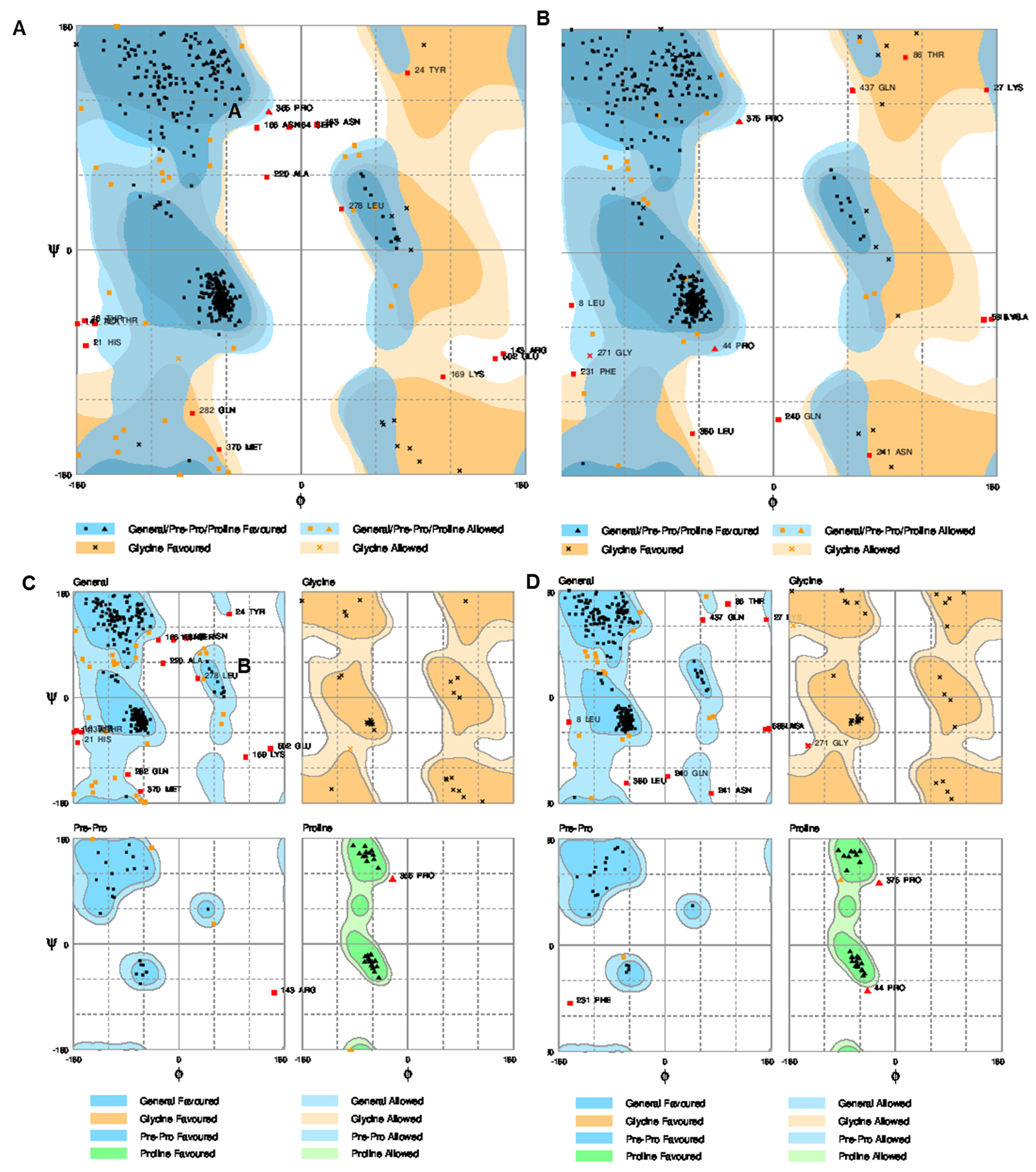

Supplement: Additional file 3: Figure S3. — Ramachandran plot of WsCYP98A and WsCYP76A 3D models of Withania somnifera using RAMPAGE server. Most favoured regions are coloured red, additional allowed, generously allowed, and disallowed regions are indicated as yellow, light yellow and white fields, respectively. [file 12896_2014_89_MOESM3_ESM.tiff]

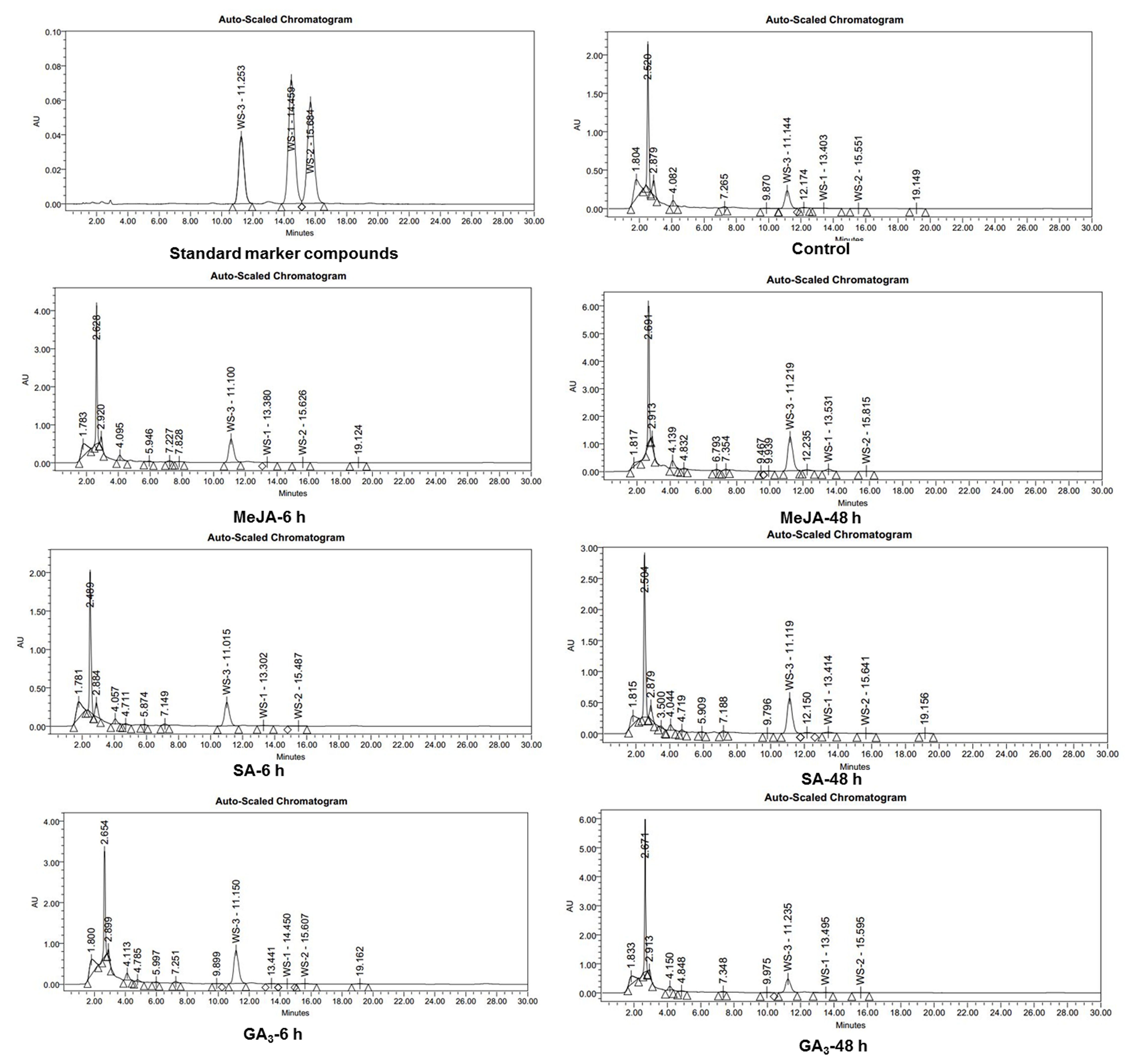

Supplement: Additional file 4: Figure S4. — HPLC analysis of withanolide A (WS-1), withanone (WS-2) and withaferin A (WS-3) at 6 h and 48 h from methyl jasmonate (0.1 mM), salicylic acid (0.1 mM) and gibberellic acid (0.1 mM ) treated micro-shoots of Withania somnifera. [file 12896_2014_89_MOESM4_ESM.tiff]
